# Retraction Note to: Bacterial conversion of depolymerized Kraft lignin

**DOI:** 10.1186/s13068-018-1307-5

**Published:** 2018-11-12

**Authors:** Krithika Ravi, Omar Y. Abdelaziz, Matthias Nöbel, Javier García-Hidalgo, Marie F. Gorwa-Grauslund, Christian P. Hulteberg, Gunnar Lidén

**Affiliations:** 1 Department of Chemical Engineering, Lund University, P.O. Box 124, 221 00 Lund, Sweden; 2 Department of Chemistry, Applied Microbiology, Lund University, P.O. Box 124, 221 00 Lund, Sweden; 3 Present Address: Australian Institute for Bioengineering and Nanotechnology (AIBN), The University of Queensland, Brisbane, QLD 4072 Australia

## Retraction to: Biotechnol Biofuels (2018) 11:240 10.1186/s13068-018-1240-7

The authors have retracted this article [1] because due to a mistake in the laboratory, SEC analyses of some of the lignin samples used had already been published as part of another article [2]. The data in the section “Characterization of depolymerized lignin streams” was therefore inaccurately presented as new, and part of the materials and methods section was incorrect. To avoid confusion this article has been retracted and the authors have been given the opportunity to resubmit their corrected data. All authors agree to this retraction.
